# CognIFied: protocol for a pilot randomised controlled trial of a culturally adapted, task-shifted compensatory cognitive training intervention for young adults with first-episode psychosis in Nigeria

**DOI:** 10.1136/bmjopen-2025-115815

**Published:** 2026-03-12

**Authors:** Abiodun O Adewuya, Adeniran Okewole, Bolanle Ola, Olabisi E Oladipo, Azizat Lebimoyo, Arit Esangbedo, Ayantunde Ayankola

**Affiliations:** 1 Lagos State University College of Medicine, Lagos, Nigeria; 2 Centre for Mental Health Research and Initiative, Lagos, Nigeria; 3 Neuropsychiatric Hospital, Abeokuta, Nigeria; 4 Department of Psychiatry, University of Cambridge, Cambridge, UK; 5 Griffith University Faculty of Health, Gold Coast, Queensland, Australia; 6 Department of Social Welfare Services, Lagos State University Teaching Hospital, Lagos, Nigeria; 7 Lagos State University Teaching Hospital, Lagos, Nigeria; 8 Federal Neuro Psychiatric Hospital, Yaba, Lagos, Nigeria

**Keywords:** Schizophrenia & psychotic disorders, Cognition, Psychosocial Intervention, Clinical Trial

## Abstract

**Introduction:**

Cognitive impairment is present in the majority of individuals with first-episode psychosis (FEP) and is a strong predictor of long-term functional disability. Despite this, evidence-based cognitive interventions are rarely available in routine mental healthcare in low-income and middle-income countries, where most young people with psychosis reside. This protocol describes the CognIFied study, a pilot randomised controlled trial evaluating the feasibility and acceptability of a culturally adapted, task-shifted compensatory cognitive training (CCT) intervention for young adults with FEP in Nigeria.

**Methods and analysis:**

CognIFied is a multicentre, assessor-blind, parallel-group pilot randomised controlled trial with an embedded mixed-methods process evaluation. The study will recruit 180 young adults aged 18–30 years with Diagnostic and Statistical Manual of Mental Disorders, Fifth Edition (DSM-5)-defined FEP (onset within the past 5 years) and objective cognitive impairment from three public psychiatric hospitals in Nigeria. Participants will be randomised 1:1 to receive either culturally adapted CCT or an active control condition, Enhanced Recreational Therapy. Both interventions comprise 12 weekly group sessions lasting 60–90 min. CCT is delivered by trained psychiatric social workers using a manualised curriculum co-designed with young people with lived experience. Primary outcomes assess feasibility (recruitment, retention, intervention adherence), acceptability (Client Satisfaction Questionnaire-8) and intervention fidelity. Secondary outcomes include preliminary signals of effectiveness on global cognitive functioning (Brief Assessment of Cognition in Schizophrenia) and functional capacity (University of California, San Diego [UCSD] Performance-Based Skills Assessment), assessed at baseline and at 3, 6 and 12 months. Quantitative analyses will be descriptive and exploratory, supplemented by qualitative inquiry guided by Reach, Effectiveness, Adoption, Implementation, Maintenance (RE-AIM) and Consolidated Framework for Implementation Research frameworks and an exploratory economic evaluation.

**Ethics and dissemination:**

Ethical approval has been obtained from relevant institutional review boards. Findings will be disseminated through open-access publications, policy-focused stakeholder engagement and community dissemination co-led by a Youth Research Team.

**Trial registration number:**

ISRCTN44794154.

**STRENGTHS AND LIMITATIONS OF THIS STUDY**

- This multicentre pilot randomised controlled trial employs an assessor-blinded, parallel-group design with allocation concealment using sequentially numbered opaque sealed envelopes, stratified by site and age group.
- The inclusion of an active control condition structurally matched on session number, duration, group size and facilitator contact minimises confounding from non-specific therapeutic factors.
- An embedded mixed-methods process evaluation, guided by the RE-AIM framework and Consolidated Framework for Implementation Research, enables systematic examination of implementation mechanisms alongside quantitative feasibility outcomes.
- Independent fidelity monitoring of a random 20% sample of audio-recorded sessions, using a structured adherence and competence checklist, provides an objective index of task-shifted delivery integrity.
- As a pilot feasibility study, the trial is not powered to detect definitive effects on clinical outcomes, and preliminary estimates of effectiveness should be interpreted with caution.

## Introduction

### Background and Rationale

 Psychotic disorders typically emerge during late adolescence and early adulthood, a critical period that often determines long-term functional trajectories.^1^ First-episode psychosis (FEP), defined as the presentation with full-threshold psychotic symptoms within the preceding 5 years, occurs with a substantial estimated incidence (34 per 100 000 persons annually).^2 3^ While hallmark positive symptoms, such as hallucinations and delusions, remain the focus of acute psychiatric intervention, they do not constitute the sole, or even the most robust, determinant of long-term recovery and community integration.^4^

A considerable body of evidence consistently indicates that cognitive impairment is a core, enduring feature of FEP, affecting more than 75% of individuals even at first presentation.^5^ These deficits traverse multiple domains, including verbal learning and memory, processing speed, executive functioning, attention and social cognition.^6 7^ Such impairments may frequently predate the onset of full-threshold symptoms and persist during symptomatic remission, robustly predicting long-term functional disability, such as unemployment and social isolation, more reliably than the severity of positive or negative symptoms.^7 8^ Consequently, enhancing cognitive functioning is increasingly recognised as a rate-limiting step in achieving holistic recovery.

### The treatment gap in low-income and middle-income countries

Approximately 80% of young individuals experiencing FEP reside in low-income and middle-income countries (LMICs), contexts where the duration of untreated psychosis is often prolonged and resource constraints are severe.^9^,^11^ In Nigeria, routine care for FEP commonly concentrates almost exclusively on pharmacological symptom stabilisation using antipsychotic medication.^12^ Although antipsychotics are essential, their efficacy in mitigating cognitive deficits is limited, and high doses may, in fact, exacerbate these issues due to anticholinergic burden.^13^,^15^ Structured cognitive interventions are virtually absent from public-sector psychiatric services, leaving the primary driver of functional disability unaddressed and contributing to a complex ‘double burden’ of biological illness and structural exclusion from socioeconomic opportunities.^10^

### Compensatory cognitive training: a pragmatic solution

While cognitive remediation is an established component of comprehensive psychosis care in high-income settings,^16 17^ traditional restorative approaches often rely on expensive computerised drills and specialised neuropsychological staff, posing a substantial barrier to scalability in resource-limited environments.^16^ Compensatory cognitive training (CCT) offers a pragmatic and low-cost alternative. Instead of attempting to restore underlying neural deficits through repetitive practice, CCT uses a ‘prosthetic’ model, teaching manualised, behavioural strategies to circumvent cognitive weaknesses.^18 19^ Participants are trained to employ external aids (eg, calendars, alarms, notes) and internal strategies (eg, mnemonics, self-talk) to efficiently manage daily tasks.^20^ Meta-analytic evidence supports the efficacy of CCT in improving functional outcomes and quality of life.^17 19^ Its manualised curriculum and suitability for group delivery position it as a strong candidate for implementation in LMICs.^17^

### The need for adaptation and task-shifting

Despite CCT’s promise, transferring Western-developed interventions directly into new cultural contexts carries the inherent risk of ‘voltage drop’; a diminished effectiveness resulting from poor cultural fit.^21^ Differences in daily cognitive demands, family structures, literacy levels and illness explanatory models necessitate rigorous cultural adaptation.^22 23^ Furthermore, the severe shortage of mental health specialists in Nigeria necessitates innovative delivery models. Task-shifting, the redistribution of tasks to non-specialist health workers, is an established WHO-endorsed strategy designed to bridge this workforce gap.^24 25^

### Study rationale

The CognIFied study aims to address these implementation challenges by systematically adapting CCT for the Nigerian context and evaluating a delivery model reliant on task-shifting to psychiatric social workers. To our knowledge, no prior trial has evaluated a culturally adapted cognitive intervention for FEP in West Africa.^25 26^ This pilot randomised controlled trial employs a Youth Participatory Action Research (YPAR) methodology to ensure the intervention is co-designed with young people.^27 28^ Ultimately, the study seeks to generate essential data on feasibility, acceptability, implementation fidelity and preliminary clinical signals, which are requisite steps for informing the design and powering of a future definitive trial.^29 30^

### Study aims and objectives

The overarching aim of the CognIFied study is to evaluate the feasibility, acceptability and preliminary clinical signals of a culturally adapted, task-shifted CCT intervention for young adults with FEP in Nigeria, compared with an active control condition. In line with international guidance for pilot trials, the primary emphasis is placed on determining whether the intervention can be delivered with adequate fidelity and sustained engagement within existing public mental health service infrastructure, rather than on hypothesis-driven tests of efficacy.

#### Primary objectives: feasibility and acceptability

- **Objective 1 (Feasibility):** To assess the feasibility of recruiting and retaining young adults with FEP into a longitudinal cognitive intervention trial across three public psychiatric hospitals. It is anticipated that recruitment of 180 participants within a 9-month enrolment period will be achievable, corresponding to the recruitment of at least 60% of eligible individuals screened. Retention of at least 70% of randomised participants at the 12-month follow-up is expected. Among participants allocated to CCT,^19^ it is anticipated that at least 75% will attend a minimum therapeutic dose, defined as nine or more of the twelve sessions.
- **Objective 2 (Acceptability):** To evaluate the acceptability of the culturally adapted CCT intervention among service users and facilitating staff. Acceptability will be operationalised through participant satisfaction scores, with the expectation that mean scores on the Client Satisfaction Questionnaire-8 (CSQ-8)^31^ will reach or exceed 24 out of a possible 32 at the 3-month assessment.
- **Objective 3 (Fidelity):** To determine whether CCT can be delivered with adequate fidelity and competence by non-specialist health workers within a task-shifted model. It is anticipated that trained psychiatric social workers will achieve at least 80% adherence to manualised content and delivery competence benchmarks, based on independent coding of a sample of audio-recorded sessions.

#### Secondary objectives: preliminary effectiveness

- **Objective 4 (Clinical signals):** To estimate preliminary effects of CCT,^19^ relative to Enhanced Recreational Therapy (ERT),^32^ on global cognitive functioning and functional capacity over a 12-month follow-up period. Participants receiving CCT are expected to demonstrate greater improvements on the Brief Assessment of Cognition in Schizophrenia^33^ composite score and the UCSD Performance-Based Skills Assessment (UPSA-N),^34^ although these analyses are exploratory in nature.
- **Objective 5 (Broader outcomes):** To explore potential effects on real-world disability, psychiatric symptom severity and quality of life, generating effect size estimates to inform future trial design.

#### Exploratory objectives: implementation and economics

- **Objective 6 (Process evaluation)**: To examine implementation processes and contextual influences on delivery, adoption and sustainability using the RE-AIM framework,^35^ complemented by qualitative inquiry with key stakeholders.
- **Objective 7 (Economic evaluation)**: To generate preliminary data on resource use and delivery costs, enabling an exploratory cost–consequence analysis to inform future scale-up considerations.

## Methods

### Study design

**Figure 1:**
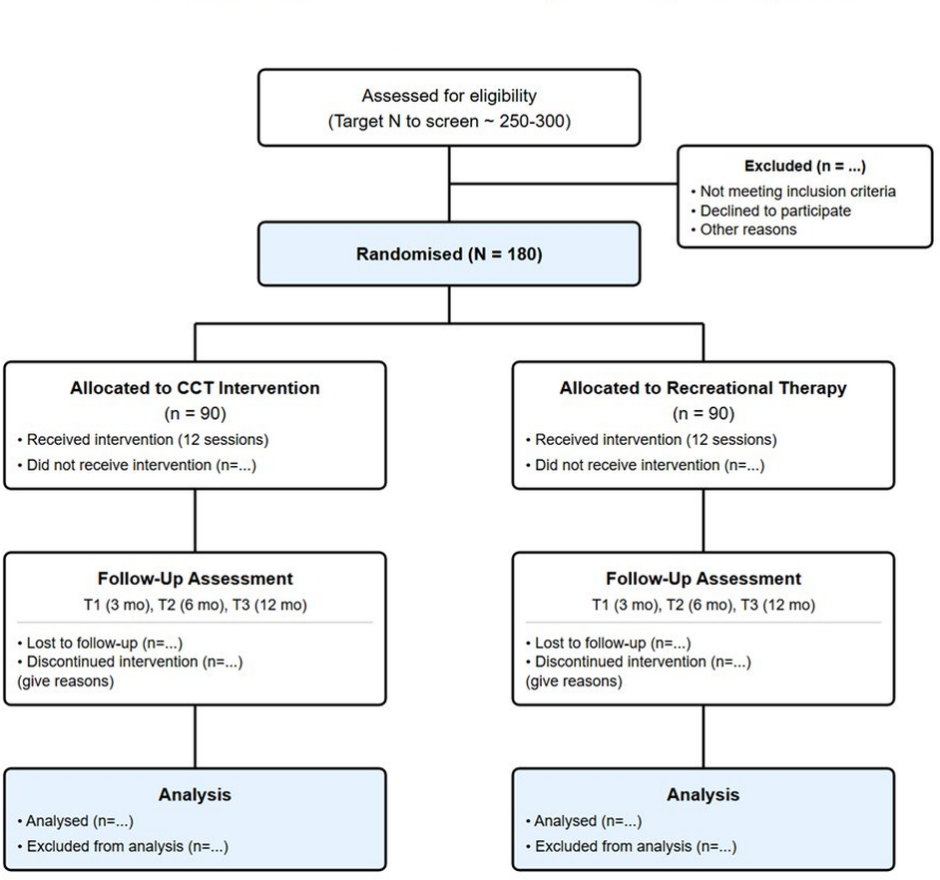
CONSORT flow diagram for the CognIFied pilot randomised controlled trial. CCT, compensatory cognitive training; CONSORT, Consolidated Standards of Reporting Trials.

The CognIFied study is designed as a multicentre, assessor-blind, parallel-group pilot randomised controlled trial (as illustrated in figure 1), with a 1:1 allocation ratio. The trial is complemented by an embedded mixed-methods process evaluation and a preliminary economic evaluation, reflecting its dual emphasis on clinical feasibility and implementation readiness. The overall design is informed by the UK Medical Research Council framework for the development and evaluation of complex interventions^36^ and is reported in accordance with the Standard Protocol Items: Recommendations for Interventional Trials 2013 guidelines^37^ and the Consolidated Standards of Reporting Trials (CONSORT) extension for pilot and feasibility trials29 (see table 1, Checklist File 1 and 2).

**Table 1:** SPIRIT schedule of enrolment, interventions and assessments

Study period	Screening	Baseline (T0)	Randomisation	Intervention period	3 months (T1)	6 months (T2)	12 months (T3)
Enrolment							
Eligibility screening (MINI 7.0)	✓						
Cognitive screening (BACS)	✓						
Informed consent	✓	✓					
Demographics and clinical history		✓					
Random allocation			✓				
Interventions							
CCT (12-weekly group sessions)				✓			
Enhanced recreational therapy (12-weekly group sessions)				✓			
Primary outcomes							
Recruitment and retention metrics	✓				✓	✓	✓
Intervention adherence (attendance)				✓	✓		
Acceptability (CSQ-8)					✓		
Intervention fidelity (audio coding)				✓			
Secondary outcomes							
Global cognition (BACS composite)		✓			✓	✓	✓
Functional capacity (UPSA-N)		✓			✓	✓	✓
Symptom severity (BPRS-18)		✓			✓	✓	✓
Disability (WHODAS 2.0)		✓			✓	✓	✓
Quality of life (MANSA, WHOQOL-BREF)		✓			✓	✓	✓
Social cognition (SCSQ-N)		✓					✓
Economic outcomes							
Resource use (CSRI)		✓					✓
Health utility (EQ-5D-5L)		✓					✓
Qualitative evaluation							
Interviews/ focus groups					✓		

BACS, Brief Assessment of Cognition in Schizophrenia; BPRS-18, Brief Psychiatric Rating Scale-18; CCT, compensatory cognitive training; CSQ-8, Client Satisfaction Questionnaire-8; CSRI, Client Service Receipt Inventory; EQ-5D-5L, EuroQol Five Dimensions Five Levels; MANSA, Manchester Short Assessment of Quality of Life; MINI, Mini-International Neuropsychiatric Interview; SCSQ-N, Social Cognition Screening Questionnaire-Nigeria; SPIRIT, Standard Protocol Items: Recommendations for Interventional Trials; UPSA-N, UCSD Performance-Based Skills Assessment; WHODAS 2.0, WHO Disability Assessment Schedule 2.0; WHOQOL-BREF, WHO Quality of Life-BREF.

**Figure 2:**
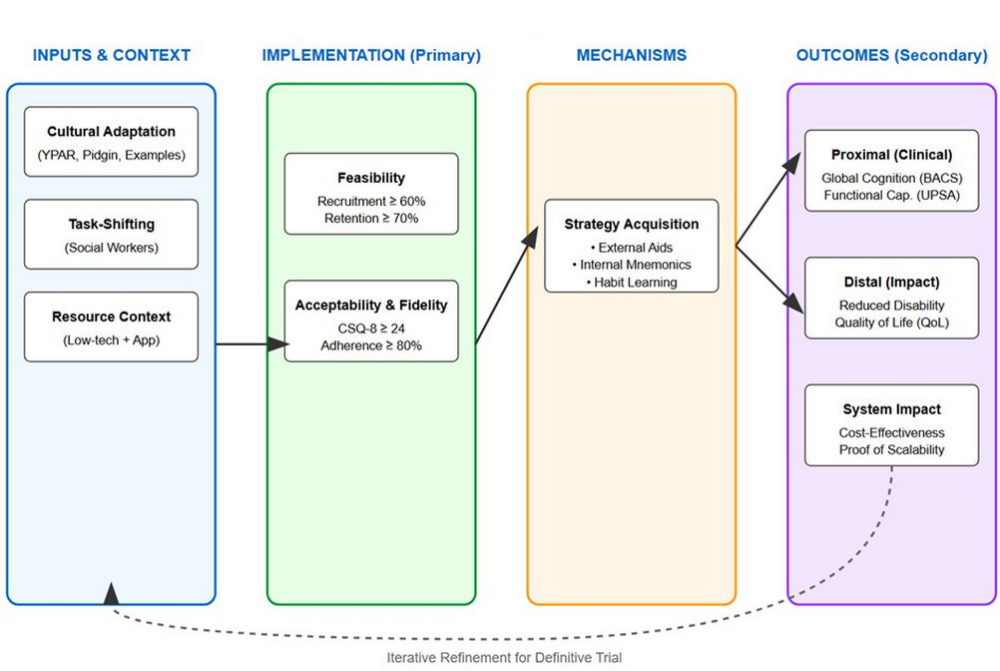
Conceptual framework linking adaptation, implementation processes, mechanisms and outcomes in the CognIFied pilot trial. BACS, Brief Assessment of Cognition in Schizophrenia; CSQ-8, Client Satisfaction Questionnaire-8; UPSA, UCSD Performance-Based Skills Assessment; YPAR, Youth Participatory Action Research.

The primary purpose of the trial is not to establish definitive clinical effectiveness, but rather to generate robust evidence on feasibility, acceptability, fidelity and preliminary outcome signals necessary to justify and inform a future definitive trial. The inclusion of a mixed-methods process evaluation allows for systematic examination of how the intervention is delivered in practice, the mechanisms through which it may exert effects, and the contextual factors that shape implementation within Nigerian public mental health services. In parallel, an exploratory health economic component is incorporated to estimate intervention delivery costs and patterns of service utilisation, supporting early assessment of scalability and affordability. The overall conceptual framework, mapping inputs, implementation metrics and mechanisms to primary and secondary outcomes, is presented in figure 2.

#### Study setting

The study will be conducted across three major tertiary public psychiatric hospitals located in Lagos and Ogun States in South-West Nigeria. These facilities were purposively selected on the basis of their high patient volumes, established outpatient services for psychotic disorders, and existing research infrastructure capable of supporting longitudinal clinical trials. Each site also employs psychiatric social workers who are embedded within routine care teams, making them suitable candidates for task-shifted delivery of the intervention.

The participating sites are:

- **Lagos State University Teaching Hospital (LASUTH), Ikeja**, a large general teaching hospital with a dedicated psychiatry department providing outpatient and inpatient mental health services.
- **Federal Neuro-Psychiatric Hospital, Yaba (FNPHY**), one of the busiest specialist psychiatric facilities in Nigeria, managing a high volume of individuals with psychotic disorders annually.
- **Neuropsychiatric Hospital Aro (NPHA), Abeokuta**, a specialist psychiatric hospital and designated WHO Collaborating Centre with longstanding involvement in mental health research and training.

Recruitment will take place primarily through outpatient clinics and early psychosis service streams within these hospitals. All intervention sessions will be delivered in existing group therapy rooms at the participating sites, ensuring that the study remains embedded within routine service environments and enhancing the ecological validity of findings.

### Participants and eligibility criteria

#### Target population

The trial will recruit 180 young adults aged 18–30 years with a diagnosis of FEP and evidence of objective cognitive impairment. Participants will be drawn from the outpatient departments of the three collaborating hospitals.

#### Inclusion criteria

Participants must meet all of the following criteria to be eligible for inclusion:

- Aged between 18 and 30 years at the time of enrolment.
- Diagnosis of a primary psychotic disorder according to DSM-5 criteria.
- Onset of first psychotic episode within the previous 5 years, confirmed using the Mini-International Neuropsychiatric Interview (MINI V.7.0).^38^
- Objective cognitive impairment, defined as a composite score at least one SD below age-adjusted and education-adjusted normative means on the Brief Assessment of Cognition in Schizophrenia (BACS).^33^
- Sufficient proficiency in English or Nigerian Pidgin English to participate meaningfully in group sessions and assessments.
- Capacity to provide written informed consent, assessed using structured teach-back methods.
- Clinical stability, operationalised as no psychiatric hospital admission within the preceding 4 weeks and no major change in antipsychotic dosage within the preceding 2 weeks.
- Current engagement in outpatient care at one of the participating study sites.

#### Exclusion criteria

Participants will be excluded if they meet any of the following criteria:

- Primary diagnosis of intellectual disability (estimated premorbid IQ <70), dementia or significant neurological disorder, including moderate to severe traumatic brain injury.
- Current substance dependence (excluding nicotine or caffeine), as defined by DSM-5 criteria.
- High and imminent risk of suicide or violence, or acute psychopathology requiring inpatient admission.
- Severe medical illness or uncorrected sensory impairment that would preclude regular attendance or participation.
- Concurrent participation in another psychosocial intervention trial or receipt of structured cognitive remediation therapy within the previous 12 months.

### Recruitment strategy

Participant recruitment is planned over a 9-month period, from 1 February 2026 to 30 November 2026, using a consecutive sampling approach. This strategy is intended to minimise selection bias while ensuring that the recruited sample reflects the clinical diversity of young people with FEP attending public psychiatric services.

Multiple, complementary recruitment pathways will be employed. Treating psychiatrists, nurses and psychiatric social workers will identify potentially eligible patients during routine outpatient visits and provide brief verbal information about the study. In addition, trained Youth Research Assistants (YRA) who are young people with lived experience of psychosis and have received training in research ethics and engagement will support recruitment by making initial approaches using culturally adapted information sheets available in English and Nigerian Pidgin English.

Eligibility screening will occur in two stages. First, diagnostic eligibility will be confirmed using the MINI V.7.0. Second, cognitive eligibility will be assessed using the BACS. Individuals who meet all eligibility criteria will be invited to provide informed consent prior to baseline assessment.

#### Informed consent procedure

Written informed consent will be obtained from all participants before any study-specific procedures are initiated. Recognising the potential impact of cognitive impairment on comprehension and decision-making, the consent process has been designed to be iterative and supportive. Research assistants will use structured teach-back methods, asking participants to explain key elements of the study, including its purpose, procedures, risks and voluntary nature, in their own words.

Participants will be given a minimum of 24 hours to consider participation and are encouraged to consult with family members or trusted individuals if they wish. Transport costs associated with screening and consent visits will be reimbursed. Only participants who demonstrate adequate understanding and voluntary agreement will be enrolled.

### Sample size and pilot rationale

The target sample size is 180 participants, with 90 allocated to each trial arm. In keeping with established guidance for pilot and feasibility trials, the sample size has been selected to provide adequate precision around key feasibility parameters rather than to ensure statistical power for hypothesis testing of clinical outcomes.

Assuming a conservative retention rate of 70%–75% at the 12-month follow-up, a sample of 180 participants allows estimation of retention with a 95% CI width of approximately ±7%. This level of precision is sufficient to apply prespecified ‘traffic light’ progression criteria to inform decisions regarding advancement to a definitive trial.

Although the study is not powered for definitive efficacy analyses, the planned sample size is expected to be sufficient to detect preliminary clinical signals. With 90 participants per arm, allowing for attrition, the study retains approximately 80% power at a two-sided alpha level of 0.05 to detect a medium effect size (Cohen’s d=0.50) on the BACS composite score. This calculation accounts for clustering effects associated with group-based intervention delivery, assuming an intraclass correlation coefficient (ICC) of 0.05.

### Randomisation and allocation concealment

#### Sequence generation

Eligible participants will be randomly assigned in a 1:1 ratio to receive either culturally adapted CCT^19^ or ERT.^32^ The randomisation sequence will be generated by an independent statistician using permuted block randomisation with varying block sizes of four, six and eight to minimise predictability. Randomisation will be stratified by study site (LASUTH, FNPHY, NPHA) and by age group (18–21, 22–26 and 27–30 years) to ensure balance across key contextual and developmental variables.

#### Allocation concealment

Allocation concealment will be maintained using sequentially numbered, opaque, sealed envelopes prepared centrally by the independent statistician. Envelopes will be stored securely at each site and opened only after completion of baseline assessments and reconfirmation of eligibility. Site-based research administrators, who are not involved in recruitment or outcome assessment, will oversee envelope opening. Recruiting staff and outcome assessors will have no access to the randomisation sequence.

#### Blinding

The trial employs an assessor-blinded design. Given the nature of the interventions, blinding of participants and facilitators is not feasible. However, all research assistants responsible for outcome assessments at 3, 6 and 12 months will remain strictly blinded to treatment allocation throughout the study period. Several procedures are in place to maintain blinding. Outcome assessors will not be involved in intervention delivery, supervision or fidelity monitoring. Assessments will be conducted in locations physically separate from intervention rooms where possible, and participants will be reminded at each assessment visit not to disclose their group allocation. To evaluate the success of blinding, assessors will be asked to guess each participant’s allocation at the 12-month assessment, and the proportion of correct guesses will be reported. Any instances of accidental unblinding will be documented and reviewed by the Data Monitoring Committee. To minimise contamination between trial arms, facilitators will be assigned exclusively to a single intervention condition, and sessions for different arms will be scheduled on separate days or times where feasible. Control arm facilitators will receive explicit instruction to avoid introducing cognitive strategies or techniques associated with the CCT curriculum.

### Interventions: culturally adapted CCT

#### Theoretical rationale

CCT, originally developed by Twamley *et al*,^19^ is grounded in a compensatory or ‘prosthetic’ model of cognitive rehabilitation. Rather than attempting to remediate underlying neurocognitive deficits through repetitive drill-based exercises, CCT focuses on teaching practical strategies that allow individuals to bypass or offset cognitive limitations in everyday functioning. This approach aligns with evidence suggesting that, in psychosis, cognitive impairments are relatively stable over time and only modestly responsive to pharmacological or restorative interventions.

CCT targets the translation of cognitive skills into real-world contexts by emphasising habit formation, environmental modification and strategic problem-solving. By prioritising functional relevance over cognitive performance per se, the intervention seeks to enhance autonomy, role functioning and participation in daily activities. This emphasis is particularly salient in early psychosis, where timely support for functional recovery may alter longer-term trajectories.

From an implementation perspective, CCT is well suited to low-resource settings. Its manualised structure and adaptability to group formats render it amenable to task-shifted delivery by non-specialist health workers, provided that adequate training and supervision structures are in place.

#### Cultural adaptation process

The CCT curriculum underwent a systematic cultural adaptation process using YPAR principles. Young people with lived experience of psychosis were engaged as co-designers throughout the adaptation phase, contributing to decisions regarding language, examples, delivery style and perceived relevance of cognitive strategies. This participatory approach was intended to enhance cultural resonance, acceptability and sustained engagement. Key adaptations included translation of materials into simplified English and Nigerian Pidgin English, with careful attention to maintaining conceptual clarity rather than literal equivalence. Western-centric examples embedded in the original curriculum were replaced with locally salient scenarios reflecting the everyday cognitive demands faced by young Nigerians, such as navigating public transportation (eg, *danfo* buses), managing informal employment or market trading, adhering to medication schedules within extended family households, and balancing religious or community obligations.

#### Intervention structure and delivery

The adapted CCT intervention consists of 12 weekly group sessions, each lasting between 60 and 90 min. A detailed overview of the curriculum content is provided in online supplemental file 1. Sessions are delivered to groups of six to eight participants to balance opportunities for peer interaction with individualised support. All sessions are conducted in designated group therapy rooms within participating hospitals, embedding the intervention within routine service settings.

Each session follows a standardised structure to promote consistency and skill consolidation. Sessions begin with a brief welcome and review of the session agenda, followed by discussion of homework assignments from the previous week. Core content is introduced through brief didactic teaching and live demonstrations, after which participants engage in guided practice exercises, including role-plays and problem-solving activities tailored to their own daily challenges. Sessions conclude with the collaborative assignment of personalised homework tasks designed to encourage real-world application of strategies.

Participants receive a printed workbook containing session summaries, worksheets and practical exercises, as well as wallet-sized cue cards highlighting key strategies.

#### Curriculum content

The curriculum targets four core neuropsychological domains commonly impaired in FEP:

1. **Prospective memory and organisation (sessions 1–2):** These introductory sessions establish a foundation of external support to alleviate the cognitive load on memory systems. Session 1: Focuses on using calendars to maintain long-term organisation and scheduling. Session 2: Introduces reminders and environmental cues to ensure tasks are completed at the appropriate time, addressing the ‘Remembering to remember’ aspect of prospective memory.
2. **Attention and working memory (sessions 3–5):** This domain transitions from external tools to the active management of information while it is being processed. Session 3: Targets short-term memory capacity, specifically the ability to retain information while simultaneously performing other actions. Session 4: Cultivates ‘Conversational Attention,’ emphasising the importance of listening well during interpersonal exchanges. Session 5: Prioritises ‘Task Attention,’ helping participants sustain focus on specific objectives to avoid distractions.
3. **Verbal learning and memory (sessions 6–8):** These modules move into the encoding and retrieval of information, leveraging specific mnemonic and organisational strategies. Session 6: Establishes the groundwork for learning new verbal information. Session 7: Teaches categorisation or ‘grouping,’ a strategic encoding technique where related items are clustered to improve recall efficiency. Session 8: Focuses on delayed memory, ensuring that information encoded previously can be successfully retrieved after a period of time.
4. **Executive function: flexibility, problem-solving and planning (sessions 9–12):** The final phase of the programme addresses higher-order cognitive control and the integration of previously learnt skills. Sessions 9 and 10: Develop cognitive flexibility, encouraging participants to ‘Think in New Ways’ and formulate a ‘Plan B’ when initial strategies fail. Session 11: Emphasises the pre-emptive phase of tasks, specifically the ability to plan a sequence of actions before execution. Session 12: Functions as a synthetic review where all compensatory skills are integrated and the programme concludes with a celebration of the mastered strategies.

Across all modules, facilitators explicitly link cognitive strategies to participants’ personally meaningful goals, reinforcing relevance and motivation. A full description of the intervention according to the Template for Intervention Description and Replication (TIDieR) checklist is available in facilitators, training and supervision: In line with the task-shifting model, CCT sessions are facilitated by psychiatric social workers rather than specialist neuropsychologists. These facilitators are already embedded within routine mental health services and possess experience in group-based psychosocial interventions.

All facilitators complete a 5-day intensive training programme prior to study commencement. Training covers the neurocognitive profile of psychosis, principles of compensatory rehabilitation, group facilitation skills and detailed familiarisation with the CCT manual. Competency is assessed through structured role-play exercises, with facilitators required to achieve at least 90% on standardised competency ratings before delivering intervention sessions.

Ongoing supervision is provided weekly by a senior clinical psychologist or psychiatrist with expertise in psychosis. Supervision sessions focus on reviewing session progress, troubleshooting challenges, reinforcing adherence to the manual and managing clinical risks. To monitor fidelity, a random 20% sample of audio-recorded sessions is independently coded using a study-specific fidelity checklist assessing both content adherence and delivery competence (see online supplemental file 2).

online supplemental file 2

### Control intervention: ERT

#### Rationale and design

The control condition employs an active control design to account for non-specific therapeutic factors such as group contact, facilitator attention, structured routine, transportation reimbursement and positive expectancy effects. This approach strengthens internal validity by allowing observed differences in outcomes to be more confidently attributed to the specific acquisition of compensatory cognitive strategies rather than to general therapeutic engagement. ERT^32^ was selected as an appropriate comparator because it provides meaningful, culturally relevant group activities without directly targeting cognitive processes or teaching structured cognitive strategies.

#### Structural equivalence

To ensure comparability, ERT is matched to the CCT intervention on key structural parameters. Participants attend 12 weekly group sessions of 60–90 min duration, delivered in groups of six to eight participants within the same hospital facilities. Sessions are scheduled with similar frequency and duration, and participants receive identical transportation reimbursement and refreshments. Sessions are facilitated by health educators or psychiatric social workers who are not involved in delivering CCT. Facilitators receive orientation to the control protocol to ensure consistency and to prevent inadvertent introduction of cognitive techniques.

#### Content and boundaries

ERT sessions focus on structured leisure and social activities designed to be enjoyable, engaging and culturally resonant. Activities include creative arts, music and cultural discussions, light physical recreation and facilitated group conversations centred on general well-being and social connection.

To preserve the conceptual distinction between trial arms, the protocol explicitly prohibits the introduction of cognitive strategies, formal problem-solving frameworks, planning techniques or memory aids. Structured homework assignments are not given. Fidelity monitoring procedures are in place to ensure that control sessions do not drift toward therapeutic content that overlaps with CCT.

#### Integrity and contamination safeguards

Several measures are implemented to minimise contamination between intervention arms. Facilitators are assigned exclusively to a single condition and do not cross-deliver sessions. Where feasible, sessions for different arms are scheduled on separate days or times. Supervision structures are kept distinct, and facilitators are regularly reminded of the importance of maintaining protocol boundaries.

### Outcome measures and assessment schedule

#### Assessment schedule

Outcome assessments are conducted at four time points by trained research assistants who remain blinded to treatment allocation throughout the study. Baseline assessments (T0) occur within 2 weeks prior to randomisation. Post-intervention assessments are conducted at 3 months following randomisation (T1; ±2 weeks), corresponding to completion of the 12-session intervention period. Further follow-up assessments are conducted at 6 months (T2; ±2 weeks) and at twelve months (T3; ±4 weeks), the latter constituting the primary endpoint for longitudinal outcomes. The schedule of enrolment, interventions and assessments throughout the study period is detailed in table 1.

This extended follow-up period is intended to capture not only immediate post-intervention effects but also the durability of cognitive and functional changes over time, an issue of particular relevance for early psychosis interventions.

#### Primary outcomes

Consistent with the pilot nature of the trial, primary outcomes focus on feasibility, acceptability and implementation fidelity rather than definitive clinical effectiveness.

- Feasibility outcomes include recruitment rate, defined as the proportion of eligible individuals who consent and are randomised; retention rate, defined as the proportion of randomised participants completing the 12-month follow-up; and intervention adherence, defined as the proportion of participants in the CCT arm attending at least nine of the 12 sessions.
- Acceptability is assessed using the CSQ-8, administered at the 3-month assessment. The CSQ-8^31^ is a widely used measure of service satisfaction, with higher scores indicating greater perceived acceptability.
- Implementation fidelity is evaluated through independent coding of a random 20% sample of audio-recorded CCT sessions. Fidelity ratings assess both adherence to manualised content and quality of delivery, providing an objective index of whether task-shifted delivery maintains intervention integrity.

#### Secondary outcomes

Secondary outcomes are included to generate preliminary estimates of effect size and variability, informing the design of a future definitive trial.

- Global cognitive functioning is measured using the composite z-score of the BACS,^33^ a validated battery assessing processing speed, verbal memory, working memory, executive function and attention.
- Functional capacity is assessed using an adapted version of the UPSA-N^34^, which evaluates participants’ ability to perform everyday tasks such as financial management, communication and planning.
- Psychiatric symptom severity is measured using the Brief Psychiatric Rating Scale-18 (BPRS-18),^39^ a clinician-rated instrument assessing positive, negative and affective symptoms.
- Real-world disability is assessed using the 12-item version of the WHO Disability Assessment Schedule 2.0,^40^ capturing functional limitations across multiple life domains.
- Quality of life is assessed using the Manchester Short Assessment of Quality of Life,^41^ supplemented by global items from the WHO Quality of Life-BREF^42^ to enhance cross-study comparability.
- Social cognition, a domain increasingly recognised as central to functional recovery, is assessed using the adapted and contextualised version of the Social Cognition Screening Questionnaire^43^ at baseline and at the 12-month follow-up only, reflecting its relative stability over shorter intervals.

#### Economic outcomes

Resource use and costs are assessed using an adapted Client Service Receipt Inventory (CSRI),^44^ capturing utilisation of formal healthcare services, informal care, non-orthodox care (including traditional and faith-based services) and productivity losses. Health-related quality of life is measured using the EQ-5D-5L^45^ to support exploratory cost–utility analyses.

A comprehensive summary of outcome domains, the specific instruments employed and their respective assessment schedules is provided in table 2.

**Table 2:** Outcome domains, instruments and assessment time points

Outcome domain	Measure	Outcome type	Assessment time points
Feasibility	Recruitment rate	Primary	Screening to randomisation
	Retention rate	Primary	3, 6 and 12 months
	Session attendance	Primary	During intervention
Acceptability	Client Satisfaction Questionnaire-8	Primary	3 months
Implementation fidelity	CCT fidelity checklist (audio-coded)	Primary	During intervention
Global cognition	Brief Assessment of Cognition in Schizophrenia composite	Secondary	Baseline, 3, 6, 12 months
Functional capacity	UCSD Performance-Based Skills Assessment–Nigeria	Secondary	Baseline, 3, 6, 12 months
Psychiatric symptoms	Brief Psychiatric Rating Scale-18	Secondary	Baseline, 3, 6, 12 months
Disability	WHO Disability Assessment Schedule 2.0 (12-item)	Secondary	Baseline, 3, 6, 12 months
Quality of life	Manchester Short Assessment of Quality of Life	Secondary	Baseline, 3, 6, 12 months
	WHO Quality of Life-BREF (global items)	Secondary	Baseline, 3, 6, 12 months
Social cognition	Social Cognition Screening Questionnaire–Nigeria	Exploratory	Baseline, 12 months
Health-related quality of life	EQ-5D-5L	Economic	Baseline, 12 months
Resource use and costs	Client Service Receipt Inventory	Economic	Baseline, 12 months

CCT, compensatory cognitive training.

### Data collection management

#### Data collection procedures

All assessments are conducted face-to-face in private consulting rooms within participating hospitals or, where necessary, at alternative agreed locations to support retention. Data are entered directly into electronic case report forms using password-protected tablets. The electronic system is hosted on REDCap, configured with built-in logic checks, range validations and audit trails to enhance data quality.

Paper-based forms are available as backup and are transcribed into the electronic database within 48 hours. Research assistants undergo rigorous training lasting 3–5 days, covering standardised assessment administration, maintenance of blinding and procedures for managing participant distress or clinical risk.

Prior to trial commencement, inter-rater reliability for clinician-rated instruments (eg, BPRS-18, UPSA-N) will be established using joint rating exercises based on standardised case vignettes and recorded interviews. ICC will be calculated, with a threshold of ≥0.80 required before independent assessment is permitted. During the study, periodic reliability recalibration sessions will be conducted every 6 months, and 10% of assessments will be independently double-rated to monitor drift. Where ICC falls below 0.75, retraining will be implemented.

#### Data security and confidentiality

All electronic data are encrypted during transmission and at rest on secure servers hosted by Lagos State University College of Medicine. Access to the REDCap database is role-based and restricted to authorised research personnel using unique login credentials. Personal identifiers are stored separately in an encrypted key file linked to study data only via unique participant identification numbers. In accordance with funder and regulatory requirements, anonymised data will be retained for a minimum of ten years following study completion.

### Quantitative data analysis

#### General analytic principles

All quantitative analyses will be conducted in accordance with a pre-specified Statistical Analysis Plan finalised prior to database lock. Analyses will be performed using Stata V.18 or R V.4.3.0. The primary analysis population is defined according to the intention-to-treat principle, with all randomised participants analysed in their assigned groups regardless of intervention adherence or withdrawal. A per-protocol analysis, restricted to participants attending at least 75% of intervention sessions, will be conducted as a sensitivity analysis.

#### Analysis of primary outcomes

Given the pilot design, analyses of feasibility and acceptability outcomes are descriptive in nature, focusing on estimation rather than hypothesis testing. Recruitment, retention, adherence and satisfaction outcomes will be summarised using proportions or means with corresponding 95% CIs. The lower bounds of these CIs will be evaluated against prespecified ‘traffic light’ criteria to inform progression decisions.

#### Analysis of secondary outcomes

Preliminary clinical signals will be explored using linear mixed-effects models to account for repeated measures over time and to accommodate missing data under a missing-at-random assumption. Models will include fixed effects for treatment group, time and their interaction, with random intercepts specified for participants and therapy groups to account for within-subject correlation and clustering.

Models will be adjusted for baseline outcome values, study site and age stratum. The primary parameter of interest is the adjusted mean difference between groups at the 12-month endpoint. Standardised effect sizes (Cohen’s d) with 95% CIs will be calculated to inform future power calculations. P values will be reported but interpreted cautiously, recognising the exploratory nature of the analyses.

#### Missing data

Patterns and predictors of missingness will be examined to assess the plausibility of the missing-at-random assumption. Where missing data exceed 15% for key outcomes, sensitivity analyses using multiple imputation by chained equations will be conducted, generating 20 imputed datasets.

#### Subgroup analyses

Exploratory subgroup analyses will examine potential effect modification by baseline cognitive severity and age group through inclusion of interaction terms in the mixed-effects models. These analyses are explicitly hypothesis-generating and will be interpreted as such.

### Qualitative process evaluation

#### Design and conceptual framework

A mixed-methods process evaluation is embedded within the trial to examine how the intervention is delivered, how participants and providers engage with it, and how contextual factors shape implementation. The evaluation is guided by the RE-AIM framework,^35^ supplemented by constructs from the Consolidated Framework for Implementation Research (CFIR)^46^ to capture multilevel influences on implementation.

##### Participants and data collection

Purposive maximum-variation sampling will be used to recruit a diverse subsample of stakeholders, including service users from both trial arms, caregivers, facilitators, supervisors and hospital administrators. Data collection methods include semistructured individual interviews and focus group discussions conducted approximately 3 months post-intervention (online supplemental file 3). Interviews and groups are conducted in English or Nigerian Pidgin by trained qualitative researchers who are independent of intervention delivery. Youth Research Team members co-facilitate selected sessions to minimise power differentials and enhance rapport.

### Data analysis

All qualitative data are audio-recorded, transcribed verbatim and anonymised prior to analysis. Framework Analysis is employed, allowing for deductive coding aligned with RE-AIM and CFIR domains alongside inductive identification of emergent themes. NVivo software is used to support data management and analysis. Qualitative findings are triangulated with quantitative data to construct an integrated implementation narrative explaining variability in engagement and outcomes.

### Health economics evaluation

#### Perspective and scope

An exploratory within-trial economic evaluation is undertaken to estimate the costs of delivering task-shifted CCT and to generate preliminary cost-effectiveness data. The primary analytic perspective is that of the public healthcare provider, with a secondary societal perspective incorporating participant and caregiver costs.

#### Measurement and valuation

Intervention costs are estimated using a micro-costing approach, capturing training, staff time, supervision, materials, facilities and digital infrastructure. Service utilisation and indirect costs are assessed using the adapted CSRI. Unit costs are derived from hospital finance departments, National Health Insurance Authority tariffs and published Nigerian health economic data. Costs are reported in 2026 Nigerian Naira and converted to international dollars for comparability.

#### Economic analysis

Given the pilot design, analyses prioritise transparency and estimation over decision rules. Cost–consequence analysis presents disaggregated costs alongside the full range of outcomes. Exploratory cost-effectiveness analyses estimate incremental cost per quality-adjusted life-year gained using EQ-5D-5L data. Uncertainty is characterised using non-parametric bootstrapping to generate cost-effectiveness acceptability curves. Details regarding the micro-costing approach and resource use categories are documented in online supplemental file 4.

### Progression criteria for a definitive trial

As a pilot feasibility study, progression to a future definitive randomised controlled trial will be guided by prespecified criteria using a structured ‘traffic light’ framework. This framework integrates quantitative feasibility metrics with qualitative insights from the embedded process evaluation, allowing progression decisions to be informed not only by whether targets are met, but also by why they are met or missed.

- **Green (Go):** Progression to a definitive trial will be recommended if recruitment rates meet or exceed 60%, retention at the 12-month follow-up meets or exceeds 70%, and intervention fidelity reaches at least 80% adherence to manualised content. Under this scenario, only minor protocol refinements would be required.
- **Amber (Amend):** If feasibility targets are narrowly missed but remain within a remediable range (eg, recruitment of 40%–59% or retention of 50%–69%), progression may still be recommended, conditional on targeted protocol modifications. Such modifications may include adjustments to recruitment pathways, increased participant reimbursement or refinements to intervention delivery identified through process evaluation findings.
- **Red (Stop):** Substantial shortfalls in feasibility (eg, recruitment below 40% or retention below 50%) will prompt a recommendation not to proceed to a definitive trial without fundamental redesign.

Progression to a future definitive trial will be guided by pre-specified 'traffic light’ criteria, integrating quantitative metrics with qualitative insights (table 3). Importantly, quantitative thresholds will not be interpreted in isolation. The Trial Steering Committee will explicitly prioritise triangulation with qualitative data to understand contextual drivers of feasibility outcomes. For example, if retention falls within the ‘Amber’ range but qualitative interviews indicate transport costs as the primary barrier, progression may still be endorsed contingent on addressing this barrier in future trial design.

**Table 3:** Progression criteria for definitive randomised controlled trial (‘traffic light’ framework)

Domain	Green (Go)	Amber (Amend)	Red (Stop)
Recruitment rate	≥60% of eligible participants randomised	40%–59% randomised	<40% randomised
Retention at 12 months	≥70% retained	50%–69% retained	<50% retained
Intervention adherence	≥75% attend ≥9 sessions	60%–74% attend ≥9 sessions	<60% attend ≥9 sessions
Acceptability (CSQ-8)	Mean score ≥24	Mean score 20–23	Mean score <20
Intervention fidelity	≥80% adherence and competence	65%–79% adherence	<65% adherence
Qualitative feasibility	No major unresolvable barriers identified	Barriers identified but remediable	Fundamental barriers to delivery

Progression decisions will be based on triangulation of quantitative metrics with qualitative process evaluation findings rather than on any single criterion alone.

CSQ-8, Client Satisfaction Questionnaire-8.

### Patient and public involvement

Young people with lived experience of psychosis were actively involved throughout the development and design of the CognIFied study. Using YPAR principles, service users contributed to the cultural adaptation of the CCT curriculum, including refinement of language, examples, delivery style and perceived relevance of cognitive strategies to everyday life in the Nigerian context.

Youth collaborators also informed recruitment materials and strategies, ensuring that study information sheets and consent processes were accessible and culturally appropriate. Trained YRAs support recruitment and retention activities, under supervision, while maintaining clear boundaries to preserve voluntariness and informed consent.

Patients and caregivers will be involved in the interpretation and dissemination of study findings through a Youth Research Team, which will co-develop community-facing summaries, infographics and stakeholder engagement activities. Participants were not involved in setting the primary outcomes of the study, which were determined based on methodological guidance for pilot trials and clinical relevance.

## Ethics, participant safety, regulatory oversight and dissemination

### Ethical approvals

The study has received full ethical approval from the Health Research Ethics Committees of all participating institutions and is conducted in accordance with the Declaration of Helsinki, International Council for Harmonisation Good Clinical Practice guidelines, and the Nigerian National Code for Health Research Ethics.

Approvals were granted by:

- Lagos State University Teaching Hospital (LASUTH): LREC/06/10/2373.
- Federal Neuro-Psychiatric Hospital, Yaba (FNPHY): FNPHY/HREC/2024/001/11/279.
- Neuropsychiatric Hospital Aro (NPHA), Abeokuta: PR/0031/25.

### Informed consent and capacity

Written informed consent will be obtained from all participants prior to enrolment. Given the presence of cognitive impairment in the study population, the consent process is deliberately iterative and supported. Research assistants will use structured teach-back methods to assess comprehension, asking participants to explain key aspects of the study in their own words before consent is accepted.

Participants aged 18–21 years provide autonomous consent in line with Nigerian legal standards. Where culturally appropriate and agreed by the participant, parallel parental or guardian awareness is encouraged, though not required. Individuals who lack decisional capacity at the time of screening due to acute symptom exacerbation are deferred until clinical stability is re-established.

#### Participant safety and adverse event monitoring

Participant safety is prioritised throughout the trial. A predefined safety protocol specifies procedures for managing participant distress, symptom exacerbation, suicidal ideation or behavioural risk. During assessments and intervention sessions, facilitators and research assistants complete a brief structured risk checklist. Participants expressing active suicidal ideation, intent or significant clinical deterioration are immediately referred to the treating psychiatrist, and same-day clinical review is arranged. All safety events are documented using a standardised adverse event form and reviewed weekly by the Trial Manager. A detailed safety protocol is provided in online supplemental file 5.

Although no significant safety risks are anticipated from either intervention, facilitators are trained to identify early warning signs of clinical deterioration and to activate appropriate referral pathways within routine care.

#### Post-trial care and access

All participants, regardless of trial arm allocation, will continue to receive routine psychiatric care at their treating hospitals following study completion. Participants who demonstrate significant clinical deterioration or adverse events during the study will be referred for appropriate clinical management within existing hospital services.

Following trial completion and pending favourable findings, the CCT intervention will be integrated into routine early psychosis services at participating sites, with all facilitators retained to support scale-up. Participants in both arms will be offered the opportunity to access CCT training materials and community-based support groups led by the Youth Research Team.

There are no anticipated harms requiring compensation beyond standard medical care. Nigeria does not have a statutory no-fault compensation scheme for research-related injury. In the unlikely event of research-related harm, participants will receive medical care through the hospital system at no additional cost.

### Data management and confidentiality

All study data are captured electronically using REDCap, hosted on secure, password-protected servers at Lagos State University College of Medicine. The database employs role-based access controls and maintains a complete audit trail. Personal identifiers are stored separately from research data in an encrypted linkage file accessible only to the Principal Investigator and Trial Manager.

Analytical datasets contain only anonymised participant identifiers. In line with funder requirements and local regulations, anonymised trial data will be securely archived for a minimum of 10 years following study completion.

### Dissemination strategy

Dissemination activities are designed to maximise both scientific and societal impact, with particular emphasis on accessibility and policy relevance.

Academic dissemination will include publication of trial findings in high-impact, open-access peer-reviewed journals regardless of outcome direction, following CONSORT guidelines for pilot trials. Conference presentations at international and regional mental health meetings are also planned.

Policy engagement will be undertaken through the development of targeted policy briefs summarising feasibility, cost implications and scalability considerations. These briefs will be shared with the Federal Ministry of Health, State Ministries of Health and relevant professional bodies to inform service planning.

Community dissemination will be co-led by the Youth Research Team, who will develop accessible summaries, infographics and short video content tailored for service users, families and the broader public. This approach reflects the study’s commitment to participatory research and knowledge democratisation.

Open science practices will be adopted. De-identified participant data will be made available to qualified researchers upon reasonable request following publication of primary findings. The culturally adapted CCT manual and training materials will be released under a Creative Commons licence to support replication and scale-up.

## Trial governance and oversight

### Trial Steering Committee (TSC)

An independent TSC provides overall oversight of the study. The committee comprises an independent chair, a psychiatrist, a biostatistician and a service-user representative. The TSC meets at least annually to review trial progress, recruitment, protocol adherence and data quality.

### Data Monitoring Committee (DMC)

An independent DMC meets every 6 months to review safety data, recruitment trends and adverse events. The DMC is authorised to recommend early termination of the trial on safety or feasibility grounds, although no formal interim efficacy analyses are planned given the pilot nature of the study.

### Protocol amendments

Any modifications to the protocol that may impact study conduct, participant safety or interpretation of results will be formally documented. Substantive amendments require prior approval from all three institutional Health Research Ethics Committees and the Trial Steering Committee. Amendments will be updated in the trial registry with version numbers, dates and rationale. Participants will be re-consented if amendments affect the risk–benefit profile or study procedures. A protocol version history log will be maintained and published alongside trial results.

## Supplementary material

10.1136/bmjopen-2025-115815 online supplemental file 1

10.1136/bmjopen-2025-115815 online supplemental file 2

10.1136/bmjopen-2025-115815 online supplemental file 3

10.1136/bmjopen-2025-115815 online supplemental file 4

10.1136/bmjopen-2025-115815 online supplemental file 5
