# Supplementary material for: CognIFied: protocol for a pilot randomised controlled trial of a culturally adapted, task-shifted compensatory cognitive training intervention for young adults with first-episode psychosis in Nigeria
Source: BMJ Open. 2026 Mar 12;16(3):e115815. doi: 10.1136/bmjopen-2025-115815 (PMC12983761; doi:10.1136/bmjopen-2025-115815)
Supplement: online supplemental file 1 [file bmjopen-16-3-s001.pdf]

CHECKLIST FILE 1

SPIRIT 2013 Checklist

Section	Item No.	Description	Addressed in manuscript
**Administrative information**			
Title	1	Descriptive title identifying the study design, population, interventions, and trial acronym	Title
Trial registration	2a	Trial identifier and registry name	Ethics, Dissemination and Oversight
	2b	All items from WHO Trial Registration Data Set	Trial registration section
Protocol version	3	Date and version identifier	Title page
Funding	4	Sources and types of financial, material, and other support	Funding Statement
Roles and responsibilities	5a	Names, affiliations, and roles of protocol contributors	Author information
	5b	Name and contact information for trial sponsor	Funding Statement
	5c	Role of study sponsor and funders	Funding Statement
	5d	Committees overseeing the trial (TSC, DMC)	Trial Governance and Oversight
**Introduction**			
Background and rationale	6a	Description of research question and justification	Introduction
	6b	Explanation for choice of comparators	Control Intervention
Objectives	7	Specific objectives or hypotheses	Study Aims and Objectives
Trial design	8	Description of trial design, including allocation ratio	Study Design
**Methods: Participants, interventions, outcomes**			
Study setting	9	Description of study settings and locations	Study Setting
Eligibility criteria	10	Inclusion and exclusion criteria	Participants and Eligibility Criteria
Interventions	11a	Detailed description of interventions for each group	Intervention Section; Supplementary File 1
	11b	Criteria for discontinuing or modifying interventions	Intervention delivery and safety
	11c	Strategies to improve adherence	Retention Strategies
	11d	Relevant concomitant care permitted or prohibited	Control Intervention
Outcomes	12	Primary, secondary, and exploratory outcomes with timing	Outcome Measures and Assessment Schedule
Participant timeline	13	Schedule of enrolment, interventions, and assessments	Table 1 (SPIRIT Schedule)
Sample size	14	Rationale for sample size	Sample Size and Pilot Rationale

Recruitment	15	Strategies to achieve adequate enrolment	Recruitment Strategy
**Methods: Assignment of interventions**			
Allocation	16a	Method for sequence generation	Randomisation and Allocation Concealment
	16b	Mechanism for allocation concealment	Allocation Concealment
	16c	Implementation of randomisation	Randomisation Procedures
Blinding	17a	Who will be blinded and how	Blinding
	17b	Circumstances for unblinding	Blinding
**Methods: Data collection, management, and analysis**			
Data collection methods	18a	Plans for assessment and data collection	Data Collection Procedures
	18b	Strategies to promote data quality	Assessor Training and Reliability
Data management	19	Plans for data entry, coding, security, and storage	Data Management and Security
Statistical methods	20a	Statistical methods for primary and secondary outcomes	Quantitative Data Analysis
	20b	Methods for additional analyses	Subgroup and Sensitivity Analyses
	20c	Definition of analysis population and missing data	Missing Data
**Methods: Monitoring**			
Data monitoring	21a	Composition and role of Data Monitoring Committee	Trial Governance and Oversight
	21b	Interim analyses and stopping guidelines	Not applicable (pilot trial)
Harms	22	Plans for collecting and managing adverse events	Participant Safety
Auditing	23	Frequency and procedures for auditing	Trial Governance and Oversight
**Ethics and dissemination**			
Research ethics approval	24	Ethics committee approval details	Ethics and Regulatory Oversight
Protocol amendments	25	Communication of protocol modifications	Ethics and Oversight
Consent	26a	Informed consent procedures	Informed Consent Procedures
	26b	Additional consent provisions	Not applicable
Confidentiality	27	Protection of participant confidentiality	Data Management and Confidentiality
Declaration of interests	28	Financial and other competing interests	Competing Interests
Access to data	29	Who will have access to final dataset	Data Management
Ancillary and post-trial care	30	Provisions for post-trial care	Ethics and Participant Safety
Dissemination policy	31a	Plans for communicating results	Dissemination Strategy
	31b	Authorship guidelines	Authorship Statement

	31c	Public access to protocol and data	Open Science Statement
**Appendices**			
Informed consent materials	32	Model consent forms and information sheets	Available on request
Biological specimens	33	Plans for collection and storage	Not applicable
