# Supplementary material for: CognIFied: protocol for a pilot randomised controlled trial of a culturally adapted, task-shifted compensatory cognitive training intervention for young adults with first-episode psychosis in Nigeria
Source: BMJ Open. 2026 Mar 12;16(3):e115815. doi: 10.1136/bmjopen-2025-115815 (PMC12983761; doi:10.1136/bmjopen-2025-115815)
Supplement: online supplemental file 2 [file bmjopen-16-3-s002.pdf]

CHECKLIST FILE 2 .

CONSORT 2010 Extension for Pilot and Feasibility Trials Checklist

Section	Item No.	Checklist item	Addressed in manuscript
**Title and abstract**	1a	Identification of the study as a pilot or feasibility randomised trial in the title	Title
	1b	Structured abstract summarising pilot trial design, methods, results (where applicable), and conclusions	Abstract
**Introduction**	2a	Scientific background and rationale for the future definitive trial, and reasons for conducting a pilot trial	Introduction
	2b	Specific objectives or research questions for the pilot trial	Study Aims and Objectives
**Methods**	3a	Description of pilot trial design (e.g. parallel, cluster) including allocation ratio	Study Design
	3b	Important changes to methods after trial commencement, with reasons	Not applicable (protocol)
**Participants**	4a	Eligibility criteria for participants	Participants and Eligibility Criteria
	4b	Settings and locations where the data were collected	Study Setting
**Interventions**	5	Description of interventions for each group with sufficient detail to allow replication	Intervention Section; Supplementary File 1
**Outcomes**	6a	Completely defined pre-specified primary and secondary outcome measures, including how and when assessed	Outcome Measures and Assessment Schedule
	6b	Any changes to trial outcomes after trial commencement, with reasons	Not applicable (protocol)
**Sample size**	7a	Rationale for sample size in a pilot trial	Sample Size and Pilot Rationale
	7b	When applicable, explanation of any interim analyses and stopping guidelines	Not applicable
**Randomisation**	8a	Method used to generate the random allocation sequence	Randomisation and Allocation Concealment
	8b	Type of randomisation; details of any restriction	Randomisation and Allocation Concealment
**Allocation concealment**	9	Mechanism used to implement the random allocation sequence	Allocation Concealment
**Implementation**	10	Who generated the allocation sequence, who enrolled participants, and who assigned participants to interventions	Randomisation Procedures
**Blinding**	11a	Who was blinded after assignment to interventions and how	Blinding
	11b	Description of similarity of interventions if blinding was not possible	Control Intervention
**Statistical methods**	12a	Statistical methods used to address pilot trial objectives	Quantitative Data Analysis
	12b	Methods for additional analyses (e.g. subgroup analyses)	Quantitative Data Analysis
**Results**	13a	Participant flow (numbers screened, randomised, followed-up, analysed)	To be reported (CONSORT flow diagram)

	13b	Losses and exclusions after randomisation, with reasons	To be reported
**Recruitment**	14a	Dates defining periods of recruitment and follow-up	Recruitment Strategy
	14b	Why the pilot trial ended or was stopped	Not applicable (protocol)
**Baseline data**	15	Baseline demographic and clinical characteristics	To be reported
**Numbers analysed**	16	Number of participants included in each analysis	To be reported
**Outcomes and estimation**	17	Results for pilot trial outcomes with measures of uncertainty	To be reported
**Ancillary analyses**	18	Results of any other analyses performed	To be reported
**Harms**	19	All important harms or unintended effects	Participant Safety
**Discussion**	20	Limitations specific to pilot trial	Discussion (future manuscript)
	21	Generalisability of pilot trial methods and findings	Discussion
	22	Interpretation consistent with pilot objectives	Discussion
**Other information**	23	Trial registration number and registry name	Ethics and Oversight
	24	Where the full trial protocol can be accessed	Main manuscript
	25	Sources of funding and role of funders	Funding Statement
	26	Ethical approval details	Ethics and Regulatory Oversight
