# Supplementary material for: CognIFied: protocol for a pilot randomised controlled trial of a culturally adapted, task-shifted compensatory cognitive training intervention for young adults with first-episode psychosis in Nigeria
Source: BMJ Open. 2026 Mar 12;16(3):e115815. doi: 10.1136/bmjopen-2025-115815 (PMC12983761; doi:10.1136/bmjopen-2025-115815)
Supplement: online supplemental file 3 [file bmjopen-16-3-s003.pdf]

CHECKLIST FILE 3

TIDieR (Template for Intervention Description and Replication) Checklist

Item No.	TIDieR item	Description as applied to the CognIFIED intervention
1	**Brief name**	*CognIFied*: Culturally adapted Compensatory Cognitive Training (CCT) for young adults with first-episode psychosis
2	**Why (rationale, theory, goal)**	CognIFied is grounded in a compensatory ("prosthetic") cognitive rehabilitation model, aiming to improve everyday functioning by teaching practical cognitive strategies that bypass cognitive impairments rather than attempting to restore underlying deficits. The intervention targets cognitive domains strongly associated with functional disability in first-episode psychosis and is adapted for delivery in Nigerian public mental health services through task-shifting to psychiatric social workers.
3	**What (materials)**	Manualised facilitator guide; participant workbooks; printed worksheets and cue cards; paper-based planners; Use of the cards for reminders and strategy practice. All materials are available in simplified English and Nigerian Pidgin English.
4	**What (procedures)**	Weekly group-based sessions incorporating didactic instruction, demonstration of cognitive strategies, guided in-session practice (e.g. role-play), and assignment of personalised homework tasks to promote real-world application. Sessions follow a standardised structure across all modules.
5	**Who provided**	Psychiatric social workers trained specifically for the study. Facilitators complete a five-day intensive training programme and receive ongoing weekly supervision from a senior clinical psychologist or psychiatrist.
6	**How (modes of delivery)**	Face-to-face, group-based delivery in hospital group therapy rooms. Sessions are interactive and participatory, with optional digital support via mobile application where available.
7	**Where**	Public tertiary psychiatric hospitals in Lagos and Ogun States, Nigeria, using existing outpatient group therapy facilities.
8	**When and how much**	Twelve weekly sessions delivered over approximately three months. Each session lasts 60–90 minutes. Groups comprise 6–8 participants.
9	**Tailoring**	The intervention is culturally adapted using Youth Participatory Action Research principles. Examples and exercises are tailored to local contexts (e.g. transportation, informal work, family routines). Facilitators personalise homework tasks to participants' daily goals.
10	**Modifications**	Minor contextual adaptations (e.g. language simplification, substitution of examples) are permitted within predefined boundaries. Any substantive deviations from the manual are documented and reviewed during supervision.
11	**How well (planned fidelity assessment)**	Fidelity is assessed through independent coding of a random 20% sample of audio-recorded sessions using a structured checklist measuring adherence and competence. A priori fidelity threshold is ≥80%.
12	**How well (actual fidelity)**	Fidelity outcomes (adherence and competence scores) will be reported descriptively in trial results. Fidelity data are used to inform supervision and to evaluate feasibility of task-shifted delivery.
