# Supplementary material for: CognIFied: protocol for a pilot randomised controlled trial of a culturally adapted, task-shifted compensatory cognitive training intervention for young adults with first-episode psychosis in Nigeria
Source: BMJ Open. 2026 Mar 12;16(3):e115815. doi: 10.1136/bmjopen-2025-115815 (PMC12983761; doi:10.1136/bmjopen-2025-115815)
Supplement: online supplemental file 4 [file bmjopen-16-3-s004.pdf]

Supplementary File 1.

Overview of the Culturally Adapted Compensatory Cognitive Training (CognIFIED) Curriculum

Purpose of this supplementary file

This supplementary file provides an overview of the structure and content of the culturally adapted Compensatory Cognitive Training (CCT) intervention evaluated in the CognIFIED trial. The purpose is to enhance transparency and replicability by outlining the session-by-session focus, core cognitive domains targeted, and key adaptations made for delivery among young adults with first-episode psychosis in Nigeria.

This document is not intended to serve as a full facilitator manual. Rather, it summarises the intervention logic and session sequencing, while detailed facilitator guidance, worksheets, and training materials are made available separately under a Creative Commons licence.

Intervention overview

CognIFIED is a 12-session, group-based Compensatory Cognitive Training programme delivered weekly over approximately three months. Sessions are designed to be delivered in groups of six to eight participants and facilitated by trained psychiatric social workers under clinical supervision.

The intervention follows a compensatory rehabilitation model, focusing on teaching practical strategies that support everyday functioning by bypassing cognitive difficulties rather than attempting to remediate underlying deficits directly. Across sessions, participants are supported to apply cognitive strategies to personally relevant goals, including medication adherence, social communication, education, work, and independent living.

The curriculum is organised to progress from foundational skills (prospective memory and attention) to higher-order cognitive functions (learning, cognitive flexibility, and planning), culminating in integration and consolidation.

Cultural adaptation principles

Adaptation of the original CCT curriculum followed Youth Participatory Action Research principles and was guided by three core considerations:

1. **Contextual relevance:** Session examples and practice exercises were reframed to reflect everyday cognitive demands faced by young people in Nigeria, including navigating public transport, informal work settings, extended family systems, and religious routines.
2. **Linguistic accessibility:** Session titles, materials, and in-session explanations were adapted into simplified English and Nigerian Pidgin English, prioritising conceptual clarity over literal translation.
3. **Resource flexibility:** Strategies were designed to be usable with or without digital tools, incorporating paper-based planners, cue cards, and optional mobile reminders to accommodate variability in access to smartphones, electricity, and mobile data.

Session structure

Each session follows a consistent structure:

- Welcome and agenda setting
- Review of previous session and homework
- Introduction of new cognitive strategy
- Guided in-session practice using role-play or real-life scenarios
- Assignment of personalised homework tasks

Sessions last between 60 and 90 minutes and are delivered in group therapy rooms within participating hospitals.

Session-by-session content overview

Session 1: Keep your plans straight (Calendars):

Introduces the programme and establishes the use of calendars as external cognitive supports. Participants practise recording appointments, medication times, and daily routines using paper or digital calendars.

Session 2: Remember things on time (Reminders and cues):
Focuses on prospective memory strategies, including lists, alarms, and linking tasks to routine daily cues to support timely task completion.
Session 3: Remember while doing something else (Short-term memory):
Addresses short-term prospective memory and divided attention. Participants practise holding information in mind while completing another task, with emphasis on everyday situations.
Session 4: Listen well (Conversational attention):
Targets conversational attention skills, including active listening, turn-taking, and paraphrasing, to support social communication.
Session 5: Stay focused (Task attention):
Builds on attention strategies to help participants reduce distractions, maintain focus, and complete tasks despite interruptions.
Session 6: Learn new words (Verbal learning 1):
Introduces verbal learning strategies such as repetition, association, and use of meaningful categories to support acquisition of new information.
Session 7: Group things that go together (Verbal learning 2):
Extends verbal learning strategies through chunking and categorisation, reinforcing generalisation to educational, vocational, and social contexts.
Session 8: Remember later (Delayed memory): Focuses on strategies to support delayed recall, including review schedules, external memory aids, and linking new information to existing knowledge.
Session 9: Have a Plan B (Flexibility 1):
Introduces cognitive flexibility and problem-solving concepts, helping participants practise generating alternative solutions when plans do not work as expected.
Session 10: Think in new ways (Flexibility 2):
Builds on flexibility skills by challenging rigid thinking patterns and encouraging adaptive responses to changing situations.
Session 11: Plan before you start (Planning):
Targets planning and organisation skills using a structured, stepwise approach to breaking down tasks, prioritising actions, and anticipating obstacles.
Session 12: Put it all together (Integration and celebration):
Consolidates skills learned across the programme, supports reflection on progress, and encourages participants to identify strategies they will continue using beyond the intervention.

Delivery and fidelity
All sessions are delivered by trained psychiatric social workers following a manualised curriculum. Fidelity is monitored through independent coding of a random sample of audio-recorded sessions using a structured fidelity checklist assessing adherence to session content and quality of delivery.

Replicability and access
The culturally adapted CognIFIED curriculum, facilitator training materials, and participant workbooks will be made freely available under a Creative Commons licence following publication of the trial results, to support replication and scale-up in other low-resource settings.

Supplementary File 2.

Compensatory Cognitive Training (CognIFIED) Fidelity and Competence Checklist

Purpose of this supplementary file

This checklist describes the criteria used to assess fidelity and quality of delivery of the culturally adapted Compensatory Cognitive Training (CognIFIED) intervention when delivered by task-shifted non-specialist facilitators. Fidelity assessment focuses on two complementary domains: **adherence** (whether prescribed content was delivered) and **competence** (how well the content was delivered). Independent raters evaluate a random sample of audio-recorded sessions using this checklist to ensure intervention integrity across sites.

Fidelity assessment overview

- **Unit of assessment:** Individual intervention session
- **Raters:** Independent psychologists not involved in intervention delivery
- **Sampling:** Random 20% of all delivered CCT sessions
- **Rating scale:** 0 = Not delivered / Poor; 1 = Partially delivered / Adequate; 2 = Fully delivered / High quality
- **Fidelity threshold:** ≥80% overall adherence and competence score

Table S2.1. Session-level adherence checklist

**Domain**	**Fidelity item**	**Rating (0–2)**
**Session structure**	Session opened with agenda review and purpose clearly stated	☐ 0 ☐ 1 ☐ 2
	Previous session and homework reviewed	☐ 0 ☐ 1 ☐ 2
	Session closed with summary and homework assignment	☐ 0 ☐ 1 ☐ 2
**Content delivery**	Core cognitive strategy for the session was introduced	☐ 0 ☐ 1 ☐ 2
	Strategy explanation aligned with manualised content	☐ 0 ☐ 1 ☐ 2
	Examples used were relevant to Nigerian context	☐ 0 ☐ 1 ☐ 2
**Skills practice**	In-session guided practice conducted	☐ 0 ☐ 1 ☐ 2
	Practice activities matched session objectives	☐ 0 ☐ 1 ☐ 2
**Homework**	Homework task assigned and clearly explained	☐ 0 ☐ 1 ☐ 2
	Homework linked to participant's daily functioning	☐ 0 ☐ 1 ☐ 2

Table S2.2. Facilitator competence checklist

**Competence domain**	**Indicator**	**Rating (0–2)**
**Communication**	Uses clear, simple, and non-technical language	☐ 0 ☐ 1 ☐ 2
	Checks participant understanding (e.g. questions, summarising)	☐ 0 ☐ 1 ☐ 2
**Engagement**	Encourages participation from all group members	☐ 0 ☐ 1 ☐ 2
	Manages group dynamics respectfully and inclusively	☐ 0 ☐ 1 ☐ 2
**Cultural responsiveness**	Adapts examples to local context appropriately	☐ 0 ☐ 1 ☐ 2
	Demonstrates sensitivity to language, literacy, and social norms	☐ 0 ☐ 1 ☐ 2
**Skill facilitation**	Models cognitive strategies accurately	☐ 0 ☐ 1 ☐ 2
	Supports participants to personalise strategies	☐ 0 ☐ 1 ☐ 2
**Boundary maintenance**	Avoids introducing non-protocol therapeutic techniques	☐ 0 ☐ 1 ☐ 2
	Maintains focus on compensatory (not restorative) strategies	☐ 0 ☐ 1 ☐ 2

Table S2.3. Global session quality ratings

Global item	Rating (0–2)
Overall adherence to session manual	☐ 0 ☐ 1 ☐ 2
Overall quality of facilitation	☐ 0 ☐ 1 ☐ 2
Session delivered within appropriate time frame	☐ 0 ☐ 1 ☐ 2
Participant engagement level	☐ 0 ☐ 1 ☐ 2

Scoring and interpretation

- **Adherence score:** Sum of all adherence items ÷ maximum possible score
- **Competence score:** Sum of all competence items ÷ maximum possible score
- **Overall fidelity score:** Mean of adherence and competence scores

Sessions achieving an overall fidelity score of **≥80%** are classified as meeting fidelity criteria. Sessions falling below this threshold are reviewed in supervision to identify training or support needs.

Use of fidelity data

Fidelity data are used for quality assurance and process evaluation rather than for participant-level exclusion. Aggregated fidelity scores are reported descriptively to characterise the feasibility of task-shifted delivery and to inform refinement of training and supervision models for future scale-up.

Supplementary File 3.

Semi-Structured Qualitative Interview and Focus Group Discussion Guides

Purpose of this supplementary file
This supplementary file outlines the semi-structured interview and focus group discussion guides used in the qualitative process evaluation embedded within the CognIFIED trial. The guides are designed to explore participant, caregiver, and provider experiences of the intervention and trial procedures, with particular attention to acceptability, perceived mechanisms of change, cultural appropriateness, and implementation feasibility.
The guides are informed by the RE-AIM framework and selected constructs from the Consolidated Framework for Implementation Research (CFIR). They are used flexibly, allowing interviewers to probe emergent issues while ensuring coverage of core domains relevant to intervention delivery and scale-up.

General interview procedures
- Interviews and focus groups are conducted approximately three months after completion of the intervention.
- Sessions are conducted in English or Nigerian Pidgin English, according to participant preference.
- All interviews are facilitated by trained qualitative researchers who are independent of intervention delivery.
- Written informed consent for audio-recording is obtained prior to data collection.
- Interviews last approximately 45–60 minutes; focus groups last 60–90 minutes.

<u>Guide A. Service user interview guide (CCT and control arms)</u>
Introduction and rapport
- Can you tell me a little about your experience of taking part in this study?
- What made you decide to join the programme?

Intervention experience and engagement (RE-AIM: Reach, Effectiveness)
- How did you find the group sessions overall?
- Which parts of the sessions stood out to you most, either positively or negatively?
- Were there any sessions or activities that you found difficult to follow or less useful?

Perceived impact and mechanisms
- Have you noticed any changes in how you remember things, pay attention, or plan your daily activities since joining the programme?
- Can you give an example of something you do differently now compared to before the programme?
- For CCT participants: Which strategies, if any, do you still use in your daily life?

Cultural relevance and acceptability (CFIR: Intervention characteristics)
- Did the examples and activities used in the sessions feel relevant to your everyday life?
- Was the language used in the sessions easy to understand?
- Were there any parts that did not fit well with your culture, beliefs, or daily routine?

Barriers and facilitators to participation (RE-AIM: Adoption, Implementation)
- What made it easy for you to attend the sessions regularly?
- What challenges did you face in attending or participating?
- How could the programme be improved to make it easier for others to take part?

Closing
- Would you recommend a programme like this to other young people with similar experiences? Why or why not?
- Is there anything else you would like to share about your experience?

<u>Guide B. Caregiver interview guide</u>
Introduction and role
- Can you tell me about your relationship with the person who took part in the study?
- How involved were you in their care during the study period?

Observed changes and impact

- Did you notice any changes in their daily functioning, memory, or organisation after the programme began?
- Were there any changes in their confidence or independence?

Family and cultural context (CFIR: Outer setting)
- How did the programme fit within your family routines and responsibilities?
- Were there any cultural or family beliefs that supported or conflicted with participation?

Acceptability and burden
- From your perspective, was participation in the programme helpful or burdensome?
- Did attending sessions or assessments create any challenges for the family?

Scalability and recommendations
- Do you think programmes like this should be part of routine care in hospitals?
- What changes would make it more acceptable or useful for families?

<u>Guide C. Facilitator focus group guide (psychiatric social workers / health educators)</u>

Introduction and role clarity
- Can you describe your role in delivering or supporting the sessions?
- How did the training prepare you for this role?

Intervention delivery and fidelity (RE-AIM: Implementation)
- How easy or difficult was it to deliver the sessions as outlined in the manual?
- Which parts of the curriculum were most straightforward to deliver? Which were more challenging?

Task-shifting experience (CFIR: Characteristics of individuals)
- How confident did you feel delivering cognitive strategies without being a specialist psychologist?
- What kinds of support or supervision were most helpful?

Contextual influences (CFIR: Inner setting)
- How did the hospital environment affect delivery of the sessions?
- Were there organisational or workload factors that supported or hindered implementation?

Sustainability and scale-up (RE-AIM: Maintenance)
- Do you think this programme could be delivered routinely in your setting?
- What would need to change for this to be sustained over time?

Closing
- What advice would you give to another hospital considering adopting this programme?
- Is there anything else you would like to add?

<u>Guide D. Key informant interview guide (supervisors and hospital managers)</u>

Strategic fit and relevance
- How does this programme align with current priorities in mental health services at your facility?
- What value do you see in addressing cognitive impairment in early psychosis?

Feasibility and resources
- What resources were most critical to implementing this programme?
- What constraints or bottlenecks did you observe?

Policy and scale-up considerations
- What would be required to scale this intervention within your institution or across other facilities?
- How do cost, staffing, and training considerations influence feasibility?

Closing
- From your perspective, what is the most important lesson from this study for future service development?

Analytic alignment

Responses from all guides are analysed using Framework Analysis, with initial coding aligned to RE-AIM and CFIR domains, followed by inductive coding to capture emergent themes. Data from different stakeholder groups are triangulated to develop a comprehensive understanding of implementation processes and contextual influences.

Supplementary File 4.

Health Economic Evaluation: Costing Framework and Resource Use Measures

Purpose of this supplementary file

This supplementary file provides additional methodological detail on the exploratory health economic evaluation embedded within the CognIFIED pilot trial. The aim is to document the costing approach, resource use categories, and analytic assumptions used to estimate the costs and potential economic consequences of delivering a task-shifted Compensatory Cognitive Training (CCT) intervention within Nigerian public mental health services.

Given the pilot nature of the trial, the economic evaluation is explicitly exploratory and intended to inform the design, data requirements, and analytic strategy of a future definitive trial, rather than to support formal cost-effectiveness decision-making.

Economic evaluation overview

The economic component of the CognIFIED study consists of a within-trial economic evaluation conducted alongside the pilot randomised controlled trial. Two analytic perspectives are adopted:

1. **Public healthcare provider perspective** (primary), capturing costs borne by the health system.
2. **Societal perspective** (secondary), incorporating participant and caregiver out-of-pocket costs and productivity losses.

The time horizon for the analysis is 12 months post-randomisation, corresponding to the longest follow-up period in the trial.

Resource use measurement

Client Service Receipt Inventory (CSRI) adaptation

Resource use data are collected using an adapted version of the Client Service Receipt Inventory (CSRI), modified for the Nigerian context through consultation with clinicians and service users. The adapted CSRI captures utilisation across four broad domains:

- **Formal healthcare services:** outpatient psychiatric visits, inpatient admissions, emergency services, medication use, and referrals to allied health professionals.
- **Informal and non-orthodox care:** visits to traditional healers, prayer camps, faith-based counselling, and other non-biomedical providers commonly accessed in the local context.
- **Participant and caregiver costs:** transportation expenses, time spent attending appointments, and direct out-of-pocket payments for care.
- **Productivity impacts:** time away from paid work, education, or vocational training for participants and caregivers.

The CSRI is administered at baseline and at the 12-month follow-up to capture changes in service utilisation over the study period.

Intervention costing approach

Micro-costing methodology

The cost of delivering the CognIFIED intervention is estimated using a micro-costing (ingredients-based) approach. This method involves identifying, measuring, and valuing all resources required to deliver the intervention under trial conditions.

Cost components include:

- **Training costs:** facilitator training time, trainer fees, training materials, venue costs, and refreshments.
- **Staff time:** facilitator time for session delivery, preparation, and documentation; supervisor time for weekly supervision sessions.
- **Materials:** participant workbooks, cue cards, printed materials, and stationery.
- **Facilities:** use of hospital group therapy rooms, valued using local estimates of space costs where available.
- **Administrative support:** scheduling, coordination, and record-keeping time.

Training costs are annualised where appropriate, assuming a conservative useful life for training of two years.

Unit cost estimation

Unit costs are derived from multiple sources to enhance accuracy and contextual relevance:

- Public sector salary scales for psychiatric social workers, supervisors, and administrative staff.
- Hospital finance department records for facility and overhead costs.
- National Health Insurance Authority (NHIA) tariff schedules for healthcare services.
- Published Nigerian health economic studies for valuation of informal care and productivity losses where direct estimates are unavailable.

All costs are initially calculated in Nigerian Naira (₦) at 2026 price levels. For international comparability, costs are converted to US dollars and international dollars using purchasing power parity (PPP) conversion factors.

Outcome measurement for economic analysis

Health-related quality of life is measured using the EQ-5D-5L at baseline and at 12 months. Utility scores are derived using available value sets, with sensitivity analyses exploring alternative scoring approaches where relevant.

Quality-adjusted life years (QALYs) are calculated using the area-under-the-curve method over the 12-month follow-up period.

Economic analysis plan

Cost–consequence analysis

Given the pilot design, the primary economic output is a cost–consequence analysis. This presents a disaggregated balance sheet comparing total costs between trial arms alongside the full range of observed outcomes, including cognitive, functional, quality-of-life, and implementation metrics. This approach avoids premature aggregation of outcomes and supports transparent interpretation by policymakers.

Exploratory cost-effectiveness analysis

An exploratory cost-effectiveness analysis estimates the incremental cost per QALY gained for CCT compared with Enhanced Recreational Therapy. Mean costs and outcomes are compared between arms, with uncertainty characterised using non-parametric bootstrapping (5,000 replications). Results are presented using cost-effectiveness planes and cost-effectiveness acceptability curves across a range of plausible willingness-to-pay thresholds.

Budget impact analysis

A simple budget impact analysis models the projected cost of scaling the task-shifted CCT intervention to all tertiary psychiatric facilities in South-West Nigeria over a five-year period. Scenarios vary assumptions regarding group size, staff mix, and session frequency to explore affordability under different implementation models.

Interpretation and limitations

Economic findings from this pilot study will be interpreted cautiously, with emphasis on feasibility of data collection, variability in costs, and identification of key cost drivers. The study is not powered to detect statistically significant differences in economic outcomes, and any cost-effectiveness estimates are considered hypothesis-generating.

Findings will be used primarily to refine the economic evaluation design of a future definitive trial and to inform early discussions with policymakers regarding scalability.
