# Supplementary material for: CognIFied: protocol for a pilot randomised controlled trial of a culturally adapted, task-shifted compensatory cognitive training intervention for young adults with first-episode psychosis in Nigeria
Source: BMJ Open. 2026 Mar 12;16(3):e115815. doi: 10.1136/bmjopen-2025-115815 (PMC12983761; doi:10.1136/bmjopen-2025-115815)
Supplement: online supplemental file 5 [file bmjopen-16-3-s005.pdf]

SUPPLEMENTARY FILE 5

SAFETY MONITORING AND ADVERSE EVENT MANAGEMENT PROTOCOL

Purpose of this supplementary file

This supplementary file provides detailed information on the safety monitoring procedures implemented in the CognIFied trial. It outlines the structured approach to risk identification, escalation pathways, classification of adverse events, reporting timelines, and documentation processes.

The purpose of this document is to enhance transparency regarding participant safety procedures in a psychosocial intervention trial involving young adults with first-episode psychosis. This document complements the safety description provided in the main manuscript and should be read alongside the trial protocol.

Safety monitoring overview

Although both Compensatory Cognitive Training (CCT) and Enhanced Recreational Therapy (ERT) are low-risk psychosocial interventions, participants may experience fluctuations in psychiatric symptoms during the study period. Safety monitoring procedures are therefore embedded within routine trial contacts and integrated with existing hospital clinical governance systems at:

- Lagos State University Teaching Hospital (LASUTH)
- Federal Neuro-Psychiatric Hospital Yaba (FNPHY)
- Neuropsychiatric Hospital Aro (NPHA)

Safety monitoring occurs during:

- Baseline assessment
- All follow-up assessments (3, 6, 12 months)
- Each intervention session
- Any unscheduled participant contact

Risk identification procedures

At each participant contact, facilitators or blinded assessors complete a brief structured safety check assessing:

- Suicidal ideation (passive or active)
- Suicidal intent or planning
- Recent self-harm behaviour
- Escalation of psychotic symptoms
- Severe agitation or aggression
- Significant medication non-adherence
- Marked functional deterioration

If no safety concerns are identified, the session or assessment proceeds as planned and documentation is completed.

If a concern is identified, severity is assessed and the escalation pathway is activated.

Escalation pathway

Low or moderate risk

Examples:

- Passive suicidal ideation without intent
- Mild symptom worsening
- Increased distress during sessions

Actions:

- Provide supportive containment
- Notify Trial Manager within 24 hours
- Inform treating psychiatrist within 24–48 hours
- Arrange routine clinical review if indicated
- Document as Adverse Event (AE), where applicable

Participants may continue trial participation if clinically appropriate.

High or imminent risk

Examples:

- Active suicidal ideation with intent or plan
- Suicide attempt
- Acute psychotic relapse requiring urgent review
- Severe agitation or violent behaviour
- Hospital admission

Immediate actions:

- Ensure participant is not left unattended
- Contact treating psychiatrist immediately
- Arrange same-day psychiatric review
- Activate emergency response if required
- Notify Principal Investigator and Trial Manager within 24 hours
- Document as Serious Adverse Event (SAE)

Continuation in the study is reviewed after stabilisation.

Figure S5.1. Risk Identification and Escalation Pathway

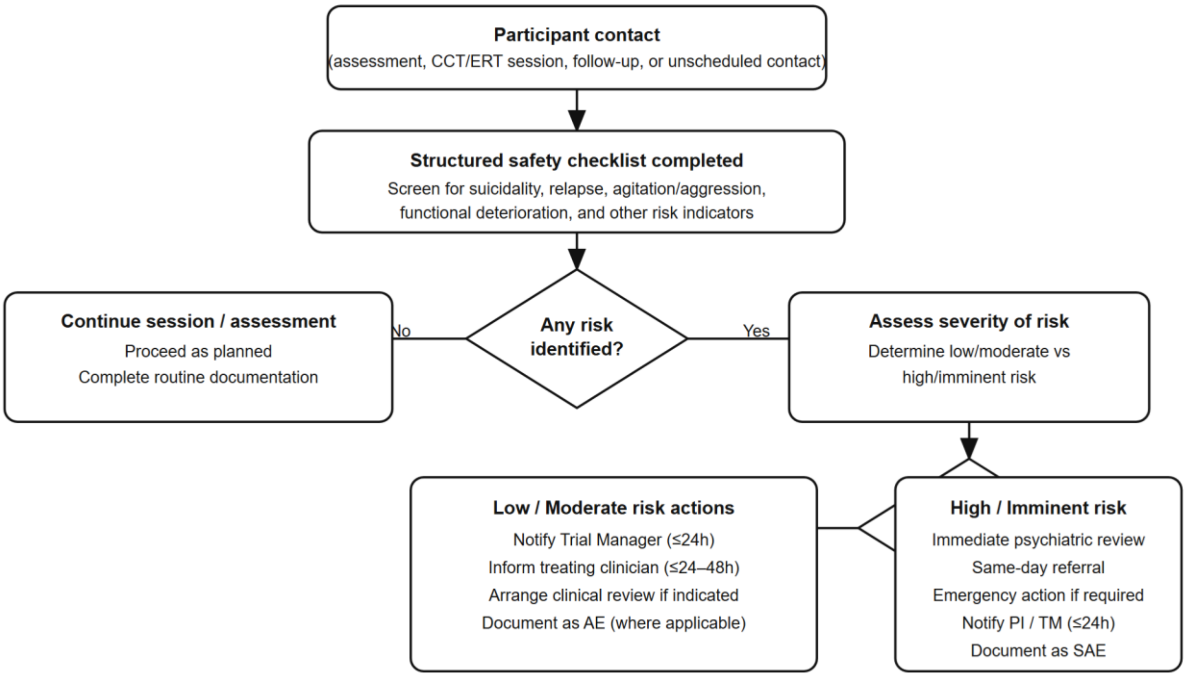

Abbreviations: AE, adverse event; SAE, serious adverse event; PI, principal investigator; TM, trial manager; CCT, compensatory cognitive training; ERT, enhanced recreational therapy.

Definitions of adverse events

Adverse Event (AE)

An AE is any untoward medical or psychological occurrence during the trial period, irrespective of attribution to the intervention.

Examples include:

- Symptom exacerbation
- Non-serious self-harm
- Medication side effects
- Increased distress during sessions
- Functional deterioration

Serious Adverse Event (SAE)

An SAE is any event that:

- Results in death
- Is life-threatening
- Requires hospital admission or prolongation of hospitalisation
- Results in persistent or significant disability
- Involves suicide attempt requiring medical intervention

Psychiatric hospitalisation for relapse is classified as an SAE.
Relationship to the intervention is recorded as:

- Not related
- Possibly related
- Probably related

Reporting timelines
Non-serious adverse events (AE)

- Recorded within 24 hours
- Reviewed weekly by the Trial Manager
- Reported in aggregate to the Data Monitoring Committee (DMC) every six months

Serious adverse events (SAE)

- Reported to the Principal Investigator within 24 hours
- Reported to relevant Health Research Ethics Committees within 24–72 hours in accordance with institutional policies
- Reported to the Data Monitoring Committee
- Included in annual trial progress reports

Documentation procedures
All adverse events are recorded using a standardised Adverse Event Reporting Form capturing:

- Participant ID (coded)
- Date of event
- Description of event
- Severity classification
- AE or SAE determination
- Attribution assessment
- Actions taken
- Outcome

Data are entered into REDCap with audit trail functionality enabled. Identifiable data are stored separately in encrypted linkage files accessible only to authorised personnel.

Oversight and review

- The Trial Manager reviews safety logs weekly.
- The independent Data Monitoring Committee reviews cumulative safety data every six months.
- The Trial Steering Committee receives annual safety summaries.

The Data Monitoring Committee may recommend protocol modification, temporary suspension, or early termination if unexpected safety concerns arise.
