# Supplementary material for: CognIFied: protocol for a pilot randomised controlled trial of a culturally adapted, task-shifted compensatory cognitive training intervention for young adults with first-episode psychosis in Nigeria
Source: BMJ Open. 2026 Mar 12;16(3):e115815. doi: 10.1136/bmjopen-2025-115815 (PMC12983761; doi:10.1136/bmjopen-2025-115815)
Supplement: Supplementary file 6 [file bmjopen-2025-115815.reviewer_comments.pdf]

PEER REVIEW HISTORY

BMJ Open publishes all reviews undertaken for accepted manuscripts. Reviewers are asked to complete a checklist review form and are provided with free text boxes to elaborate on their assessment. These free text comments are reproduced below.

ARTICLE DETAILS

Title (Provisional)

CognIFied: Protocol for a pilot randomised controlled trial of a culturally adapted, task-shifted compensatory Cognitive training Intervention for young adults with First-episode psychosis in Nigeria

Authors

Adewuya, Abiodun O; Okewole, Adeniran; Ola, Bolanle; Oladipo, Olabisi E; Lebimoyo, Azizat; Esangbedo, Arit; Ayankola, Ayantunde

---

VERSION 1 - REVIEW

**Reviewer**	**1**
**Name**	**Barrera, Alvaro**
**Affiliation**	**University of Oxford, Psychiatry**
**Date**	**21-Feb-2026**
**COI**	**None**

Thank you. This is a well written manuscript on a very important topic. I wonder whether the authors could briefly explain how the inter-rater reliability will be monitored prior to and during the study. Could they also describe the predefined safety protocol, perhaps as supplementary material?

VERSION 1 - AUTHOR RESPONSE

REVIEWER 1

We are grateful for the reviewer's positive appraisal of the manuscript and thoughtful suggestions.

Comment 1

I wonder whether the authors could briefly explain how the inter-rater reliability will be monitored prior to and during the study.

Response

We appreciate this important suggestion. We have now expanded Section 2.10 (Data Collection Management) to provide a detailed description of inter-rater reliability procedures. Specifically, we have clarified that:

- Inter-rater reliability for clinician-rated measures (e.g., BPRS-18, UPSA-N) will be established prior to trial commencement using joint rating exercises and standardised case materials.
- Intraclass correlation coefficients (ICC) will be calculated, with a minimum threshold of ≥0.80 required before independent rating is permitted.
- During the study, periodic recalibration sessions will be conducted every six months.
- A random 10% sample of assessments will be independently double-rated to monitor rater drift.
- Retraining procedures will be initiated if ICC falls below predefined thresholds.

Comment 2
Could they also describe the predefined safety protocol, perhaps as supplementary material?
Response
We thank the reviewer for highlighting the importance of greater clarity regarding safety procedures.
We have addressed this in two ways:

1. **Main Text Revision:**
   Section 3.3 (Participant safety and adverse event monitoring) has been expanded to include a clearer summary of the predefined safety protocol, including structured risk checks, escalation procedures, and documentation processes.
2. **New Supplementary File:**
   We have created a new supplementary document:

Supplementary File 5: Safety Monitoring and Adverse Event Management Protocol
This file includes:

- A structured risk identification framework
- A detailed escalation pathway
- Definitions of adverse events (AE) and serious adverse events (SAE)
- Reporting timelines
- Documentation procedures
- A visual flowchart (Figure S5.1) illustrating the escalation pathway

This addition enhances transparency regarding participant safety governance in the context of a psychosocial intervention trial involving individuals with first-episode psychosis.
